# Application of stem cells in regeneration medicine

**DOI:** 10.1002/mco2.291

**Published:** 2023-06-17

**Authors:** Ye Jin, Shuangyang Li, Qixuan Yu, Tianli Chen, Da Liu

**Affiliations:** ^1^ School of Pharmacy Changchun University of Chinese Medicine Changchun Jilin China

**Keywords:** bone regeneration, exosomes, nanoformulations, skin regeneration, stem cells

## Abstract

Regeneration is a complex process affected by many elements independent or combined, including inflammation, proliferation, and tissue remodeling. Stem cells is a class of primitive cells with the potentiality of differentiation, regenerate with self‐replication, multidirectional differentiation, and immunomodulatory functions. Stem cells and their cytokines not only inextricably linked to the regeneration of ectodermal and skin tissues, but also can be used for the treatment of a variety of chronic wounds. Stem cells can produce exosomes in a paracrine manner. Stem cell exosomes play an important role in tissue regeneration, repair, and accelerated wound healing, the biological properties of which are similar with stem cells, while stem cell exosomes are safer and more effective. Skin and bone tissues are critical organs in the body, which are essential for sustaining life activities. The weak repairing ability leads a pronounced impact on the quality of life of patients, which could be alleviated by stem cell exosomes treatment. However, there are obstacles that stem cells and stem cells exosomes trough skin for improved bioavailability. This paper summarizes the applications and mechanisms of stem cells and stem cells exosomes for skin and bone healing. We also propose new ways of utilizing stem cells and their exosomes through different nanoformulations, liposomes and nanoliposomes, polymer micelles, microspheres, hydrogels, and scaffold microneedles, to improve their use in tissue healing and regeneration.

## Graphical abstract

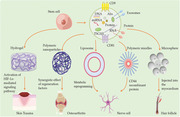

## 1 INTRODUCTION

Regenerative medicine is an interdisciplinary field that activates endogenous stem cells in the body or implants exogenous stem cells, stem cell‐derived cells, or functional tissues and organs.^1^ It repairs, replaces, and enhances damaged, diseased, or defective skin, bone, and organs in the human body to achieve disease treatment.^2^ Stem cells are primitive, undifferentiated cells with the capacity of self‐replication, multidirectional differentiation, and homing potential and also retain the properties of their parents.^3^ According to the physiological requirements of the body, stem cells can differentiate into various cell types, such as nerve cells, cardiomyocytes, and liver cells, which are necessary for tissue regeneration.^4^, ^5^, ^6^ Stem cells are subdivided into totipotent, multipotent, and unipotent stem cells according to their differentiation potential^7^; among them, pluripotent stem cells exhibit significant tissue repair capacity.^8^, ^9^ Stem cells have anti‐inflammatory properties, promote epithelial cell proliferation, and inhibit wound scarring.^10^, ^11^ In addition, stem cells and their exosomes have significant effects on bone tissue and nervous system regeneration.^12^, ^13^ Therefore, researchers have envisioned the application of stem cells in regenerative medicine to promote skin and bone tissue regeneration. However, due to the difficulties of stem cell transportation and preservation, as well as their tumorigenicity and the difficulty of exosome extraction, nanoformulations of stem cells have been studied and have gradually become a hot research topic for elucidating new ways to repair damaged skin and bone.

As the largest organ in the body, the skin plays a protective role against external damage.^14^ Owing to its external exposure and weak self‐healing ability, skin regeneration faces great challenges.^15^, ^16^ Skin regeneration is a complex and dynamic process^17^, ^18^ and generally consists of three phases: inflammatory, proliferative, and remodeling.^19^, ^20^, ^21^ The whole process is relatively long and can have a negative psychological impact on the patient.^22^ Large, chronic, and hard‐to‐heal skin wounds caused by severe trauma or radiation burns^23^ cause physiological dysfunction of the body that is prone to further infection,^24^ seriously affecting the quality of life of patients.^25^, ^26^ Moreover, the healing of bone injury is a major concern in current medical research. In the United States alone, 100,000 fractures result in nonhealing bone injuries annually.^27^ The bone plays an important role in supporting movement and protecting organs and is one of the essential organs for maintaining life activities.^28^, ^29^, ^30^, ^31^ Although the bone is a highly vascularized organ with a certain ability of regeneration, the external damage beyond its self‐healing range results in nonhealing and scarring of the bone tissue.^32^ In such cases, recovery is difficult through autologous bone tissue regeneration alone, and the healing time is increased; therefore, patients experience pain.^33^ Therefore, a new treatment method is needed to promote skin and bone tissue regeneration.^34^

The first controlled‐release formulation was introduced in 1950. Significant advances in nanodrug delivery technology^35^ and the continuous development of nanotechnology^36^ have led to its application in the field of drug delivery systems.^37^ Nanoformulations can selectively target drug transport to fingertip sites, thus reversing the traditional drug delivery process and enabling higher biocompatibility,^38^ safety, eco‐friendliness, specificity, and reduced toxicity while maintaining therapeutic efficacy.^39^ In addition, nanoformulations can cross the cellular barrier and activate the transport mechanism.^40^ Therefore, drug activity can be maintained to the maximum extent,^41^ ensuring fast and stable drug action. When used in skin regeneration, combination with biologically active molecules prevents the drug from being degraded by proteases in the wound, thus improving drug stability.^42^ The direct use of easily degradable and metabolically fast stem cells and their exosomes and the use of nanoformulations in skin and bone tissue healing is of great importance for improving the use of stem cells and their exosomes in regenerative medicine. Several studies have shown that nanoformulations such as hydrogels and liposomes can significantly improve the speed and time of bone healing with the use of drugs.^43^, ^44^

In this paper, we attempt to contribute to the field of regenerative medicine by reviewing the main mechanisms of stem cells and their exosomes for tissue regeneration and the progress of novel nanoformulations loaded with stem cells and exosomes in the field of tissue regeneration.^4^ We also describe the modification effect of genetic engineering in stem cells and their exosomes to in the regeneration of skin and bone tissue.

## 2 STEM CELLS IN REGENERATIVE MEDICINE

In continuous research, the position of stem cells in the field of regenerative medicine have gradually become a hot research topic.^45^ Stem cells are widely used in regenerative medicine research because of their strong self‐renewal ability and ease of extraction.^46^ Some stem cell treatments have already been in clinical trials, especially for skin and bone regeneration.^47^, ^48^, ^49^

### 2.1 Introduction to stem cells

Since the 20th century, stem cell and regenerative medicine technology have been one of the hot frontiers in the international biomedical field, which plays an irreplaceable role in safeguarding human life and health in improving the quality of human survival and extending human life expectancy.^50^ Stem cells are undifferentiated cells characterized by a high capacity for proliferation and self‐renewal as well as clonality usually derived from a single cell and differentiated into different types of cells and tissues, these properties may vary between stem cells.^51^ For example, embryonic stem cells (ESCs) obtained from blastocysts and adult humans have different properties, with ESCs from blastocysts having higher tissue specificity. According to the stage of stem cell development, ESCs and adult stem cells (ASCs) are classified.^52^ ESCs can self‐regenerate and differentiate into all tissues in vivo. Numerous animal studies have shown that ESCs promote neural regeneration in neuron‐deficient and chronically denervated rat models.^53^ However, the application of ESCs is currently limited due to ethical issues. In addition to this, adult stem cells include cells present in tissues or organs that have the potential for further differentiation, the ability to self‐renew, and the ability to differentiate into the major types of specialized cells of the tissue from which they originate. ASCs mainly include mesenchymal stem cells (MSCs), myogenic stem cells (MDSCs), neural stem cells (NSCs), and urogenic stem cells (USCs).^54^

MSCs are one kind of adult stem cells that originate from the early mesoderm and self‐renewing mesodermal cells with multidirectional differentiation potential, which can be isolated from adult bone marrow, dental pulp, adipose tissue, umbilical cord, and other tissues.^55^ Among the stem cell family, MSCs are the most widely studied and used stem cells in the field of regenerative medicine. According to different sources, they can be divided into: adipose‐derived MSCs, umbilical cord MSCs, bone marrow MSCs, and so on.^56^ There are relevant research tables showing that it has good application prospects in the field of regenerative medicine, and some MSCs have already entered clinical application.^57^ MSCs have many advantages such as easy isolation, high in vitro expansion, low immunogenicity, and targeted differentiation into neural tissue cells.^57^ MDSCs have long‐lasting self‐renewal and multigerm differentiation ability and can differentiate not only into mesodermal myoblasts, osteoblasts, chondrocytes, adipocytes, endothelial cells, hematopoietic cells, but also into ectodermal neuronal cells.^58^ With immunity, donor cells can still be detected weeks after the acute inflammatory response and the damaged nerve can still be functionally regenerated after transplantation of human MDSCs.^59^ NSCs transplantation provides a new therapeutic idea for neurological injury diseases and promotes the development of neural regeneration. NSCs not only have the function of cell replacement, but also play the function of immunomodulation, and regulate the activation of complement.^60^ Dental stem cells (DSCs) are a population of embryonic neural crest‐derived adult stem cells that are homologous to neural tissue.^61^ DSC has stronger proliferation and multidirectional differentiation ability than MSC from other tissues and is one of the ideal stem cell sources for tissue regeneration because it is easy to obtain with features of less invasive and less immune rejection.^62^ Urine‐derived stem cells are new stem cells with good expansion capacity, multilineage differentiation potential, and paracrine function.^63^ Increasing interests in this aspect shows that it can be isolated from urine samples with the advantages of easy collection, noninvasive sampling, and few ethical issues.^64^

Stem cell therapy represents an important trend in the development of medicine for regeneration. The role of stem cell therapy in repairing, restoring or reconstructing human tissue and organ damages is becoming more and more prominent. The application of stem cells and their derivatives has solved many medical problems in recent years. Skin and bone injuries often lead to severe disability, and exploration of promising therapeutic strategies is of great importance. As the largest organ in the body, the skin is vulnerable to injury and weak in self‐repair. Furthermore, the bones are vulnerable to injury causing greater damage, relying solely on self‐repair cycles that leading to more pain on patients. These reasons making skin and bone a current research hotspot in regenerative medicine.^47^, ^48^, ^49^

### 2.2 Stem cells promote skin tissue regeneration

MSCs were first observed in the bone marrow in 1867 by Kornheim, who found that these cells may be the source of fibroblasts involved in wound repair.^65^ Studies have confirmed that stem cells play an important role in the treatment of various skin injuries^66^ and numerous scholars have studied their regenerative repair mechanism.^67^, ^68^ Additionally, stem cells can effectively suppress the inflammatory response and assist in wound repair for skin regeneration through their immunomodulatory effects (Table 1). In this article, we discuss the use of stem cells in several stages of wound healing.

**TABLE 1:** Stem cells applied to skin and bone regeneration and their mechanism advantages.

Type	Mechanism	Dominance	Model	References
Adipose‐derived stem cells (ADSCs)	Participation in immune regulation Through paracrine effect Inhibit scar formation	Rich source Small damage Large quantity Strong proliferative capacity Low immunogenicity	Cellular models Rheumatoid arthritis Mouse model	^72^, ^88^
Umbilical cord‐matrix stem cells (UCMCs)	Control inflammatory response Promoting granulation angiogenesis and cell proliferation Inhibit scar formation	Donor risk‐free Rapid and simple Painless Noninvasive sampling Low potential culture cost	Diabetic mice	^75^
Bone marrow mesenchymal stem cells (BM‐MSCs)	homing features Multidirectional differentiation Immune regulation and immunosuppression Paracrine effect Increased anti‐inflammatory expression	Easy to obtain simple culture technology Low immunogenicity	Critical limb ischemia rats model Deep partial‐thickness burns rats model Collagen‐induced arthritis murine model diabetic mice Cranial defect rat model Bone damage in rats	^75^, ^76^, ^85^, ^86^, ^89^, ^90^

The inflammatory phase plays a crucial role in the wound healing process by inducing the recruitment of immune cells to prevent continued pathogenic damage to the organism.^69^, ^70^ During the hemostatic phase, stem cells migrate to the local wound site and induce vasoconstriction and platelet agglutination to promote blood clotting.^71^ To produce hemostatic and anti‐inflammatory effects, a closed wound is temporarily formed on the wound surface.^72^ A few hours after wound formation, the inflammatory phase begins, which is characterized by local congestion and plasma exudation. Some studies have used flow cytometry to examine cell proliferation and demonstrated that MSCs have potent immunomodulatory effects and possess antimicrobial properties by regulating the functional properties of T cells, B cells, and dendritic cells.^73^ In the early stages of inflammation, the level of wound inflammatory factors increases, including the anti‐inflammatory factors interleukin (IL)‐4, IL‐10, IL‐13, and others. In an experiment on rats, MSCs increased the level of anti‐inflammatory factors, whereas that of proinflammatory factors and tumor necrosis factor (TNF)‐α, gamma‐interferon, IL‐1, IL‐6, IL‐8, intercellular adhesion molecule‐1, and others was decreased.^74^

Macrophages play a major immunomodulatory role after 3 days of wound formation and are the key regulators of the local inflammatory microenvironment. After injecting the wound surface of diabetic mice with MSCs, it was found that macrophages were recruited to the wound in large numbers, which in turn induced macrophages to acquire the M2 anti‐inflammatory phenotype.^75^ This caused the wound to produce an immune response that inhibited bacterial and other undesirable proliferation and promoted tissue regeneration. Another reported further studies that by experimenting with mouse bone marrow MSCs. Assessment of the immunosuppressive effects of different immune cells. MCSs promote macrophage polarization while inhibiting B‐cell differentiation by regulating IL‐1 receptor antagonists.^76^ The anti‐inflammatory effects of MSCs have been well illustrated. Furthermore, MSCs induce the production of IL‐10 by macrophages at inflammatory sites. IL‐10 is a cytokine with anti‐inflammatory properties and induces regeneration of traumatized tissue, which greatly contributes to wound healing and lays the foundation for wound repair.

During the entire repair process, recruited stem cells produce paracrine factors. These paracrine factors exhibit antiapoptotic, proangiogenic, and accelerated cell proliferation effects, which are necessary to promote tissue regeneration.^77^, ^78^ Exogenous MSCs implanted in the injured areas can produce cytokines, such as fibroblast growth factor (FGF)‐2, hepatocyte growth factor (HGF), vascular endothelial growth factor (VEGF), and transforming growth factor (TGF)‐β through paracrine effects.^79^ These cytokines promote angiogenesis as well as fibroblast migration and proliferation.^80^ Moreover, they accelerate the deposition of collagen,^81^ regulate the inflammatory response in damaged skin tissues, and promote skin tissue regeneration.^82^

Reportedly, treatment using paracrine factors (TGF‐β, FGF‐2, angiopoietin‐2, and VEGF‐1) secreted by transplanted bone marrow MSCs after myocardial infarction modeling in rats manipulated the microenvironment and reduced inflammation.^83^ From this experiment, it can be concluded that the cytokines produced by stem cells at the infarct site can effectively promote angiogenesis and accelerate cell migration. In addition, a study conducted on mouse spinal cord MSCs by coculturing dermal fibroblasts with insert‐grown bone marrow MSCs and cell scratching experiments showed that the paracrine effect of bone marrow‐derived MSCs could accelerate the proliferation and migration of dermal fibroblasts, apart from the chemotaxis assay.^84^ The migration of endogenous MSCs and endothelial progenitor cells has also been considered by researchers as an important mechanism for repairing damaged skin. Moreover, a study reported that MSCs of bone marrow and adipose origin could be isolated from a rat model of severe limb ischemia.^85^ In vitro and in vivo experiments revealed that bone marrow‐derived MSCs can promote endothelial cell migration, muscle reorganization, limb function improvement, and neovascularization. In addition, a previous study on rats has reported treatment of deep second‐degree burns with bone marrow MSCs through paracrine effects via the stromal cell‐derived factor (SDF)‐1 alpha/CXCR4 pathway.^86^ This approach induces the accumulation of endogenous MSCs and endothelial progenitor cells at the wound site, which can accelerate skin healing. Many of the therapeutic capabilities of stem cells rely on their paracrine actions. Various growth factors regulate the differentiation of fibroblasts and endothelial progenitor cells and promote their transfer to the wound. This mechanism promotes the formation of new blood vessels and granulation tissue, laying the foundation for skin regeneration.^36^ Stem cell paracrine factors acting on damaged skin tissue are more effective than direct stem cell differentiation^87^ and the healing is faster.^87^ The potential clinical applications of cytokines derived from stem cells and the experimental models mentioned in the literature are listed in Table 2.

**TABLE 2:** Potential clinical application fields of stem cell active factors.

Stem cell active factor	Mechanism	Potential clinical application	Target organ	Model	References
PDGF (platelet‐derived growth factor)	Anti‐inflammatory immune regulation and angiogenesis	Burns skin repair	Vascular regeneration	Burn‐rats model	^36^
VEGF (vascular endothelial growth factor) SDF‐1 (stromal cell‐derived factor‐1)	Promote Mature ECs Migration Promoting angiogenesis and artery formation	Treatment of acute myocardial infarction, chronic ischemic heart disease, and congestive heart failure	Myocardial regeneration Vascular regeneration	Deep partial‐thickness burns rats model	^80^
VEGF‐1 (vascular endothelial growth factor‐1)	Inducing angiogenesis in ischemic tissue Angiogenesis induced by proliferation of endothelial cells and vascular smooth muscle cells Stimulate endothelial cells to sprout from existing blood vessels	Treatment of myocardial infarction Improve myocardial function Increase angiogenesis in infarcted area	Tumor microenvironment Embolization site	Animal and preliminary human	^83^
Ang (angiogenin)	Improving capillary density of ischemic tissue Promote the formation of new blood vessels	Ischemic disease of hind limbs	Ischemic tissue	Critical limb ischemia rats model	^85^
SDF1‐α (stromal cell‐derived factor 1 alpha)	Enhance angiogenesis Reduce apoptosis	Burns Skin repair	Vascular regeneration	Deep partial‐thickness burns rats model	^86^

### 2.3 Stem cells promote bone tissue regeneration

Bone is a mineralized tissue that provides structural support to the vertebrate body.^91^ It protects internal organs, attaches to muscles to allow the body to move, and plays an important role in regulating serum calcium and phosphorus levels.^92^ The response of bone tissue to trauma is a continuous and complex process that includes an inflammatory response, activation of repair mechanisms, and remodeling of various tissues.^93^ If a bone defect is followed by delayed healing or nonhealing that develops into a larger defect, traditional restorative means are no longer sufficient to restore such defects.^94^, ^95^ Regenerating damaged bone tissue to its predisease state has been a major challenge for clinicians and researchers worldwide.^96^ Researchers aim to address this problem with new treatments.^97^ Recently, stem cells have been found to be a good medium and option for achieving bone tissue regeneration.^98^ The use of bone marrow MSCs in bone tissue engineering has entered the preclinical phase, and the U.S. Food and Drug Administration has approved an in vitro cell manufacturing procedure for obtaining biologically active bone marrow MSCs from high‐quality human bone marrow.^92^ Stem cells can migrate to the site of injury and have the ability to differentiate into local components of the injury site and help regenerate the tissue.^99^, ^100^, ^101^, ^102^ For example, Kaku et al.^89^ isolated MSCs from the bone marrow of rat femurs and transplanted them into a rat model with cranial defects. These results suggest that MSC transplantation may be a new option for cranioplasty. MSCs can not only repair bone tissue but also form suture‐like gaps and promote cranial bone growth.

A direct reflection of the initial stage of bone injury is the formation of hematoma and the onset of an inflammatory response; hematoma can effectively prevent excess bleeding.^103^ The fracture hematoma is initially infiltrated by immune cells, mainly neutrophils and macrophages.^104^ When the bone tissue is damaged, blood vessels rupture, platelets are activated, and multiple coagulation factors, cytokines, and chemokines are released, triggering a hemostatic effect. Multiple cell types are recruited to migrate to the site of injury, contributing to the initial stage of bone tissue repair.^105^ Inflammation is a protective response of tissues against harmful stimuli and leads to the initiation of the healing process.^106^, ^107^ Transplantation of stem cells into bone injury sites revealed the systemic anti‐inflammatory effects of the cytokines they release.^108^ The acute inflammatory phase peaks at 24−48 h after injury and subsides after one week. On the first day of a bone injury, neutrophils arrive at the injury site and release signaling molecules that recruit macrophages to the injury site.^109^ Granero‐Molto et al.^108^ reported the presence of bone marrow MSCs in the region of new bone formation in a mouse fracture model. This indicated the promoting effect of stem cells on bone tissue regeneration. Macrophages not only engulf necrotic cells and tissue debris at the fracture site, but also initiate the recruitment of MSCs and vascular progenitor cells.^110^, ^111^ During acute inflammation, macrophages amplify the inflammatory response by phagocytosing invading microbes and recruiting additional immune cells to restore tissue homeostasis.^112^ A study demonstrated that MSCs can mediate elevated M1 macrophage marker expression and promote M2 macrophage polarization, tissue vascularization, and bone volume increase in a rat femoral defect model.^113^ This study illustrated the anti‐inflammatory and osteogenic effects of stem cells. Furthermore, it was found that BMSCs reversed the pure Lap‐induced polarization of mouse‐derived macrophages RAW 264.7 cells from M1 to M2 and promoted osteogenesis.^114^ These results suggested that BMSCs improved Lap‐induced inflammation and enhanced bone formation. BMSCs were isolated from rats and cocultured with macrophages using hydrolyzed fish collagen, which inhibited the proliferation of PBMCs and significantly increased the expression levels of anti‐inflammatory mediators, including IL‐6, TGF‐β1, and PGE2.^115^ BMSCs exhibit immunosuppressive capacity through crosstalk with immune cells, significantly limiting the inflammatory response after bone injury.^116^ Inflammatory cytokines play important role in regulating bone tissue destruction and promoting bone tissue regeneration.^90^ Bone healing after an injury is a complex biological and biomechanical process,^117^ and final healing is highly dependent on the initial inflammatory phase, which is influenced by local and systemic responses to the injury stimulus. In addition, immune cells and mesenchymal stromal cells are involved in critical intercellular communication and crosstalk to regulate bone healing. Therefore, understanding the mechanisms of stem cells in bone healing is important for practical application.

It has been found that during the repair process, recruited stem cells secrete various chemokines, cytokines, and growth factors, which are collectively known as paracrine factors and are necessary to promote tissue repair and regeneration or tissue differentiation.^65^, ^77^, ^87^ Cytokines produced by stem cells through their paracrine action, including IL‐1, IL‐6, platelet‐derived growth factor (PDGF), VEGF, and bone morphogenetic protein (BMP), play an essential role in bone tissue regeneration.^118^ These cytokines initiate the process of bone tissue formation and generate new blood vessels.

A study conducted by transplanting stem cells to an injured site revealed that the stem cells significantly reduced IL‐6 levels on days 1 and 3 after the fracture, and reduced TNF‐α and IL‐1β levels on day 3.^119^ This process inhibits tissue damage and prevents the development of fibrosis, thereby promoting rapid bone tissue regeneration. In our previous study, we reported that the cytokines secreted by BMSCs treated with sinusoidal electromagnetic fields transplanted into a rat cranial defect model could better promote neoangiogenesis and bone immunomodulation, which play important roles in bone regeneration.^120^ The osteogenic potential of BMSCs and the facilitative role of the paracrine function of BMSCs in promoting bone regeneration were illustrated. Pearson et al.^121^ implanted BMP‐2 in a rat femoral defect model, which significantly and strongly induced neovascularization, and found BMP‐2‐induced angiogenesis through paracrine signals secreted by bone progenitor cells. The presence of cytokines such as insulin‐like growth factor‐1 (IGF‐1), VEGF, TGF‐β1, and HGF in MSC‐CM significantly increased the migration and expression of genes associated with osteogenesis, and bone regeneration was observed in the rat cranial bone.^122^ In addition to traditional methods, some emerging cytokine techniques are gradually being developed. For example, Yu et al.^123^ evaluated the bone regeneration effect of collagen membranes chemically coupled to SDF‐1α in a rat model and found the chemical coupling to significantly promote the formation of new bone and microvasculature compared with the physical adsorption of SDF‐1α. The study also provided a cell‐free method to shorten bone healing time and improve the success rate of guided bone regeneration.

### 2.4 Other applications of stem cells in regenerative medicine

Apart from thoughts mentioned above, stem cells can promote regeneration of nerve and hair follicle tissues in addition to skin and bone tissues. Central nervous system diseases, such as stroke, traumatic brain injury, Alzheimer's disease (AD), and Parkinson's disease, are usually accompanied by neuroinflammation and nerve regeneration.^124^ Nerve injury is one of the most common types of traumatic injury, commonly occurring in accidents and medically induced injuries.^125^ However, traditional treatments are limited, which helps to find an alternative therapy is especially critical, and stem cell therapy is an excellent medium. Studies have shown that transplantation of stem cells are highly effective, both in terms of the number of axons and the restoration of function, showing the safety of this method.^126^ In a short period of injury, the myelin sheath of the distal nerve dissociates, macrophages infiltrate and phagocytic debris produce an inflammatory response. Yang et al.^127^ found that transplantation of undifferentiated tooth‐derived stem cells (DSCs) could reduce the inflammatory response and promote neuronal regeneration by inhibiting the expression of IL‐1 and member A of the Ras homologue gene family. Moreover, it has been found that transplanted NSCs reduce M1‐type proinflammatory macrophage infiltration, promote M1‐type to M2‐type polarization, increase the release of anti‐inflammatory factors (e.g., IL‐10), and exert neuroprotective effects.^128^ After stem cell transplantation, the stem cells are still able to produce and secrete various immune factors and neurotrophic factors to improve neurological function, even if they do not differentiate directly into nerve cells or mature nerve cells differentiated after limited transplantation.^129^ Nerve regeneration requires the growth of nerve axons and secreted expression of neurotrophic factors, such as FGF‐2, GDNF, and BDNF. A significant increase in the number of synapses was found after transplantation of stem cells to the injury site.^130^ Studies have shown that stem cells are effective in treating neurological disorders, significantly promoting changes in neurological function.^131^ Vojnits et al.^132^ suggests transplantation of injury‐induced MDSCs or their cell extracts into Duchenne muscular dystrophy mice (i.e., mdx mice) significantly promotes morphological and functional recovery of already injured neuromuscular junctions. In addition, umbilical cord MSCs (UC‐MSCs) have multidirectional differentiation possibilities and improve cognitive dysfunction caused by AD after transplantation.^133^ Stem cells for neural regeneration have a wide range of applications, but there are still many challenges for their clinical application, such as the lack of gold standard for cell isolation and culture, and preservation methods, genetic material variation and ethical issues. However, it is believed that these problems will be solved as the research progresses.

Stem cells are also a research hotspot in the field of hair follicle regeneration. Hair follicle is an organ with self‐renewal ability and under normal conditions. The hair growth cycle goes through three phases—anagen phase, apoptosis phase, and resting phase to maintain the cycle for life.^134^ However, due to the weak physical resistance of hair follicles and their susceptibility to genetic or environmental factors, leading to irreversible damage to hair follicles. Therefore, hair follicle regeneration has become an extremely important research area in the field of regenerative medicine. The role of stem cells in promoting hair follicle regeneration has become a hot issue.^135^ Under normal physiological conditions, stem cells in each region maintain their own tissue renewal. When trauma occurs, stem cells located in the augmentation site can rapidly divide and proliferate to participate in the repair of the hair follicle.^136^ ASCs are isolated from adipose tissue and induced to differentiate in dermal papilla formation medium for 30 days by visual and histological observation. The results of growth factor secretion were evaluated by immunohistochemistry and immunoblotting, showing that ASCs dermal papilla‐like tissues (DPLTs) have characteristics of dermal papillae and have a positive effect on hair regeneration by secreting growth factors.^137^ Stem cells can produce bioactive factors such as VEGF, IGF, and PDGF through paracrine action. When stem cells are applied to damaged hair follicles, these substances can regulate the hair growth cycle and promote hair growth.^138^ Bu et al.^139^ conducted a study in which cord blood MSCs were isolated and cultured in vitro and induced to differentiate into CK15 hair follicle cells. High speed induction of high purity differentiation into hair follicle cells was achieved by adding hair follicle extracellular matrix (ECM) to the culture medium. This study provides a new idea to promote the regeneration of hair follicle tissue and has a wide range of application prospects. And Fukuoka et al.^140^ reported that ADSCs were isolated from the adipose tissue of healthy women, and the supernatant of ADSCs was collected and made into a lyophilized powder to act on the damaged hair follicles of the patients, and after 1−3 months of treatment, the number of hair follicles in the patients increased significantly. A previous study reported that mouse‐derived pluripotent stem cells were effective in promoting hair follicle development.^141^ Stem cells have a broad application prospect in hair follicle regeneration since the development of regenerative medicine.

### 2.5 Clinical applications of stem cells

Regenerative medicine refers to the use of biological and engineering methods to promote repair and regeneration of damaged tissues, which restores the normal tissue characteristics and functions of an organism. The application of stem cells in regenerative medicine has gained widespread attention over the last 50 years. Because of their strong self‐renewal and dividing abilities, stem cells are the most widely used cells in the field of tissue regeneration therapy and play an important role in tissue healing and regenerative medicine.^142^ According to our research, bone marrow MSCs, umbilical cord MSCs, and adipose MSCs have shown good ability to promote skin tissue regeneration.^88^ Hassan, a 7‐year‐old Syrian “butterfly” boy, underwent treatment using his own skin cells to replace almost all his skin^143^ and has since gained new life (Table 3). Stem cells have low immunogenicity and can be allografted or xenografted for clinical treatment. Exogenous stem cells are implanted to promote wound repair for skin regeneration.

**TABLE 3:** Clinical applications of stem cells in tissue regeneration.

Type/intervention/treatment	Diseases/conditions	Phase	Trial number	References
Bone marrow stem cells (BMC)	Diabetic foot	Phase 2	NCT01065337	^47^
Adipose‐derived stem cells (ADSCs)	Diabetic foot Venous ulcer Pressure ulcer Diabetic foot ulcer	Phase 2	NCT02092870 NCT03754465	^48^, ^49^
Autologous bone marrow stem cells (BMC)	Type IV pressure Ulcers Chronic wounds Spinal cord injury	Phase 2	NCT01572376	^146^
Mesenchymal stem cells (MSCs)	Diabetic foot Lower limb ischemia	Phase 1	NCT02304588	^147^
Umbilical cord mesenchymal stem cells (UC‐MSCs)	Difficult to healing of skin ulcers	Phase 1	NCT02685722	^58^
Stem cell	Chronic wounds Pressure sores Hematopoietic stem cells Wound healing	Phase 1	NCT00535548	^148^
Injection the mesenchymal stem cell in nonunion site	Nonunion fracture	Phase 2	NCT01788059	^149^
Biological: Application of autologous mesenchymal stem cells	Mandibular fractures	Phase 3	NCT02755922	^150^
Biological: Mesenchymal stem cells Other: culture medium without MSC	Nonunion fracture	Phase 2	NCT01429012	^151^
Biological: MSC	Nonunion fracture Metaphyseal fibrous defect	Early Phase 1	NCT02307435	^152^

Bone is a highly vascularized organ, and the skeletal system responds to external stimuli in numerous ways.^144^ Repairing bone defects has always been an important clinical challenge, and innovations and improvements in bone repair are changing daily. Compared with traditional means of tissue repair, regenerative cellular and molecular techniques are improving, and the repair effect of stem cells on tissues is demonstrated by the ability to induce and stimulate differentiation into parenchymal cells in vitro to replace damaged areas.^4^, ^145^ There have been several clinical trials on stem cells for bone regeneration. Thus, the promotion of stem cell paracrine action on bone tissue regeneration is illustrated, and it is noteworthy that the paracrine function of stem cells has recently received more attention than the direct differentiation of stem cells.

## 3 STEM CELL EXOSOMES IN REGENERATIVE MEDICINE

In 1983, Pan et al.^153^ studied the maturation process of transferrin receptors in sheep reticulocytes in vitro and for the first time discovered that cells could release tiny extracellular vesicles (EVs), which were named exosomes in 1987.^154^ Exosomes are a class of membranous vesicles that contain biologically active molecules such as lipids, nucleic acids, and proteins. They are secreted extracellularly through a specialized mechanism known as endo‐and exocytotic vesicles, with a diameter of approximately 30−100 nm.^155^ They are the smallest known EVs and are secreted by almost all living cells.^156^, ^157^ Exosomes can be separated from various body fluids, such as urine and blood. if this communicates your intended meaning.^97^, ^158^ Exosomes are present in the culture medium of most cells.^159^

Stem cells produce exosomes in a paracrine manner. Stem cell exosomes are safer and more effective than stem cells with similar biological properties. Additionally, cell transplantation is also not required in this method. Stem cell exosomes represent a new way to achieve tissue regeneration, repair, and accelerate wound healing. Studies have shown that MSC‐derived exosomes (MSC‐Exos) can function well in different environments. MSC‐Exos induced repair in mouse models of wound healing and myocardial infarction.^160^, ^161^, ^162^ EVs of MSCs are rich in MSC‐derived bioactive molecules and regulate the phenotype, function, and homing of immune cells. Application of MSC‐EVs has a significant inhibitory effect on the inflammatory response of an organism, accelerating the survival and regeneration of damaged parenchymal cells.^163^ Human umbilical cord MSCs‐derived exosomes are effective in preventing cardiac dysfunction caused by aging. UMSC‐derived exosomes inhibit the NF‐κB/TNF‐α signaling pathway by releasing novel lncRNAs,^164^ thus preventing aging‐induced cardiac dysfunction. MicroRNA‐342‐3p has a regulatory role in MSC‐Exos against breast cancer, confirming that microRNA‐342‐3p inhibits metastasis and chemoresistance of cancer cells by targeting ID.^165^ Exosomes of stem cells can transfer proteins and DNA molecules between cells via paracrine or endocrine signals; therefore, the whole biological process is changed.^166^ MSC exocytosis delivers healing proteins and RNA to recipient cells,^167^ thus adding to the vitality of the therapy. The delivery of functional substances from exosomes to receptor cells helps to heal injured or diseased tissues and organs. MSC‐Exos can be manipulated to establish a new cell‐free therapeutic approach and can be used to heal skin wounds.^168^

### 3.1 Stem cell exosomes promote skin regeneration

Similar to stem cells, stem cell exosomes play different roles at different stages of wound healing. Exosomes can promote cell proliferation and neovascularization by regulating the inflammatory response^169^ (Table 4). In addition, they can inhibit scarring and promote other mechanisms of effective skin tissue regeneration.^170^, ^171^, ^172^

**TABLE 4:** Mesenchymal stem cell exosome‐acting signaling pathways promote skin tissue regeneration.

Type	Signaling pathways	Dominance	References
Bone marrow mesenchymal stem cells exosomes (BMSC‐Exos)	Activation of the PTEN/AKT signaling pathway	Inhibits proinflammatory phenotype M1‐type polarization Promote macrophage anti‐inflammatory phenotype M2‐type polarization	^173^
Molecular mechanism of human umbilical cord MSC‐derived exosomes (hUCMSC‐Exos)	Inhibition of TLR4 signaling pathway	Increase the expression level of anti‐inflammatory factors	^174^
Adipose‐derived stem cells exosomes (ADSC‐Exos)	Activation of PI3K/AKT signaling pathway Activation of Wnt/β‐catenin pathway	Affects MMP‐2 and TIMP‐1 protein expression Mediates h2o2‐induced wound healing	^175^, ^176^

The inflammatory response is the initial stage of skin tissue regeneration can produce an immune response that includes proliferation of immune cells together with secretion of inflammatory and chemotactic factors.^177^ A previous study reported that diabetic mice wounded by molding were treated by implantation of exogenous MSC exosomes. Exosomes were found to activate the PTEN/AKT signaling pathway to promote macrophage anti‐inflammatory phenotype M2‐type polarization and inhibited proinflammatory phenotype M1‐type polarization. Furthermore, they inhibited the expression of proinflammatory factors IL‐1β and TNF‐α, promoting the expression of the anti‐inflammatory factor IL‐10, which accelerated the healing rate of skin injury in mice. This effectively demonstrated their ability to promote skin tissue regeneration.^173^ Several investigators have reported that human exosomes derived from umbilical cord MSCs can mediate miR‐181c expression and were found to effectively inhibit the TLR4 signaling pathway.^174^ Exosomes can increase the expression of anti‐inflammatory factors, which, in turn, reduce the inflammatory response to burn‐induced skin damage in rats. Several researchers have conducted studies in which MSC‐Exos were found to act as recruiters and trainers of immune cells, synergistically promoting the levels of beneficial macrophages and regulating T cells in the skin wounds of mice. They not only inhibited the proliferation of T lymphocytes but also promoted the conversion of activated T lymphocytes to regulatory T cells.^178^ This inhibits the inflammatory reaction and promotes skin tissue regeneration. A moderate inflammatory response plays a role in fighting infection and removing damaged cells as well as broken cell debris; however, an excessive inflammatory response can prolong the healing cycle of skin wounds, with serious consequences.^179^ Therefore, reducing the inflammatory response during wound healing is an important aspect of skin wound repair.^180^

As the inflammatory response proceeds, there is a gradual transition from the inflammatory phase to the proliferative phase, in which leukocytes release cytokines, approximately 3−10 days after injury. This results in the induced migration of endothelial cells and fibroblasts to the wound center as well as the formation of new blood vessels and granulation tissue. The results of a study using MSCs in diabetic rats showed increased expression of CD31 and Ki67 in whole‐skin wounds. This contributed to the enhanced regenerative capacity of granulation tissue and increased expression levels of VEGF and TGFβ‐1.^181^ In addition, it made wound closure significantly faster and effectively promoted proliferation of skin tissue cells. Several studies have shown that miR‐21 is highly expressed in the exosomes of adipose MSCs and can activate the PI3K/AKT signaling pathway to affect MMP‐2 and TIMP‐1 protein expression.^175^, ^176^ This significantly promotes accelerated wound healing. It enhances the proliferation of keratinocytes and fibroblasts while promoting the production of granulation tissue. Moreover, MSC‐Exos were reported to significantly increase the level of angiopoietin 2 in wounds and enhance angiopoietin 2 proliferation, migration, and tube‐forming abilities.^182^ This mechanism suggests that stem cell exosomes play an important role in the proliferative phase of skin tissue regeneration.

The last stage of skin regeneration is the remodeling phase, which is mainly characterized by a gradual decrease in the production of new blood vessels and gradual fibrosis of the granulation tissue. During this process, fibroblasts proliferate significantly, and the newly deposited collagen molecules cross‐link, leading to an increase in the tensile strength of the tissue, which results in scar remodeling to restore the normal skin structure, a phase that can last for months to years.^183^ In particular, the therapeutic effect of TSG‐6‐modified MSC‐Exos was investigated in a mouse total wound model. The results demonstrated that exosomes from TSG‐6‐modified BMSCs inhibit scar formation by reducing inflammation and inhibiting collagen deposition.^184^ According to a previous report, treatment of mice with umbilical cord‐derived MSC‐Exos was found to increase the density of myofibroblasts and inhibit myofibroblast accumulation after a period of time.^185^ They have also been shown to reduce the somatic inflammatory response and scarring. Stem cell exosomes reduce scarring during the remodeling phase of skin tissue regeneration, mainly by inhibiting the accumulation of fibroblasts and suppressing collagen deposition.^186^, ^187^, ^188^

**FIGURE 1:**
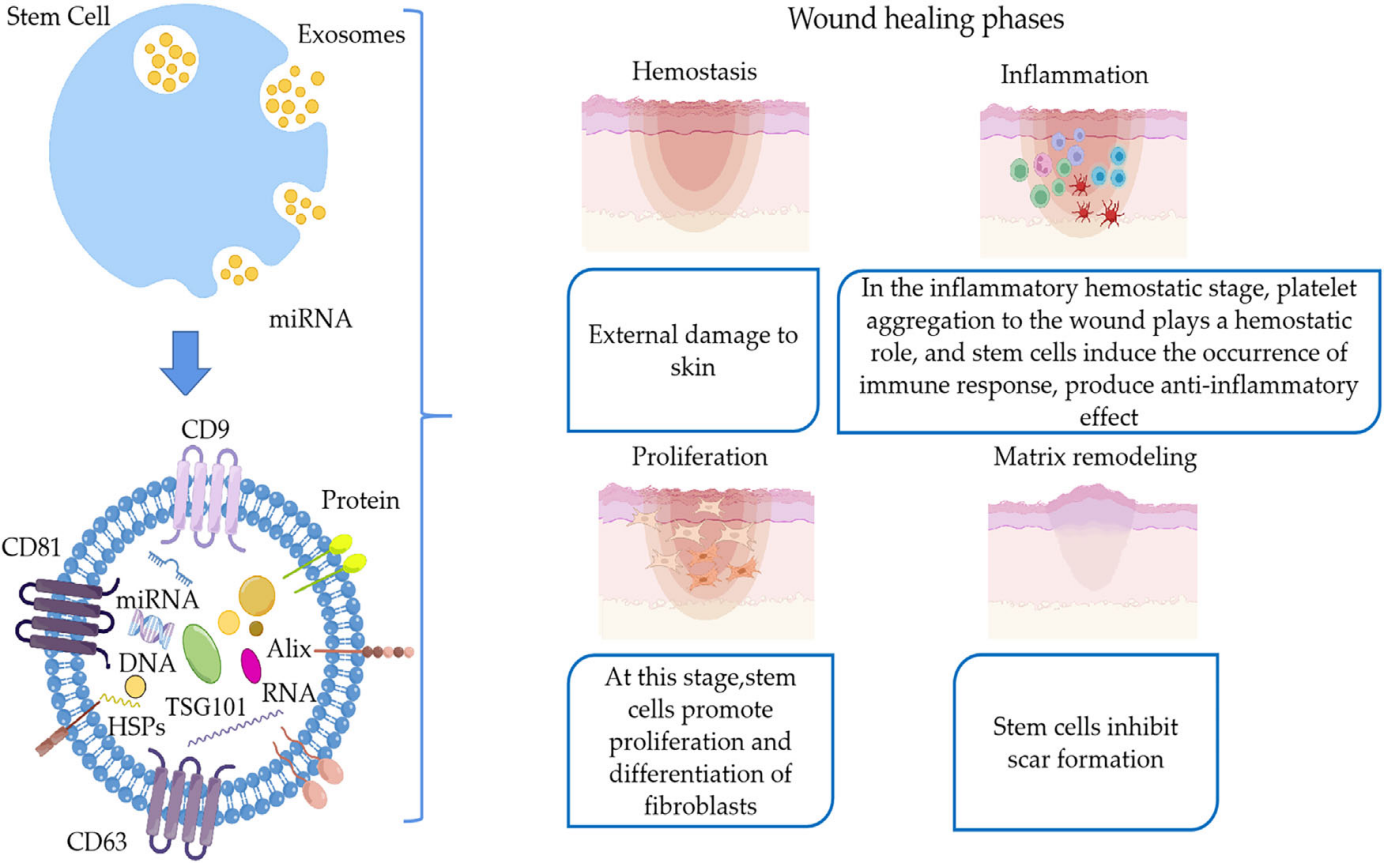
Stem cells, exosomes, and the role of stem cells in each step of the wound healing process.

**FIGURE 2:**
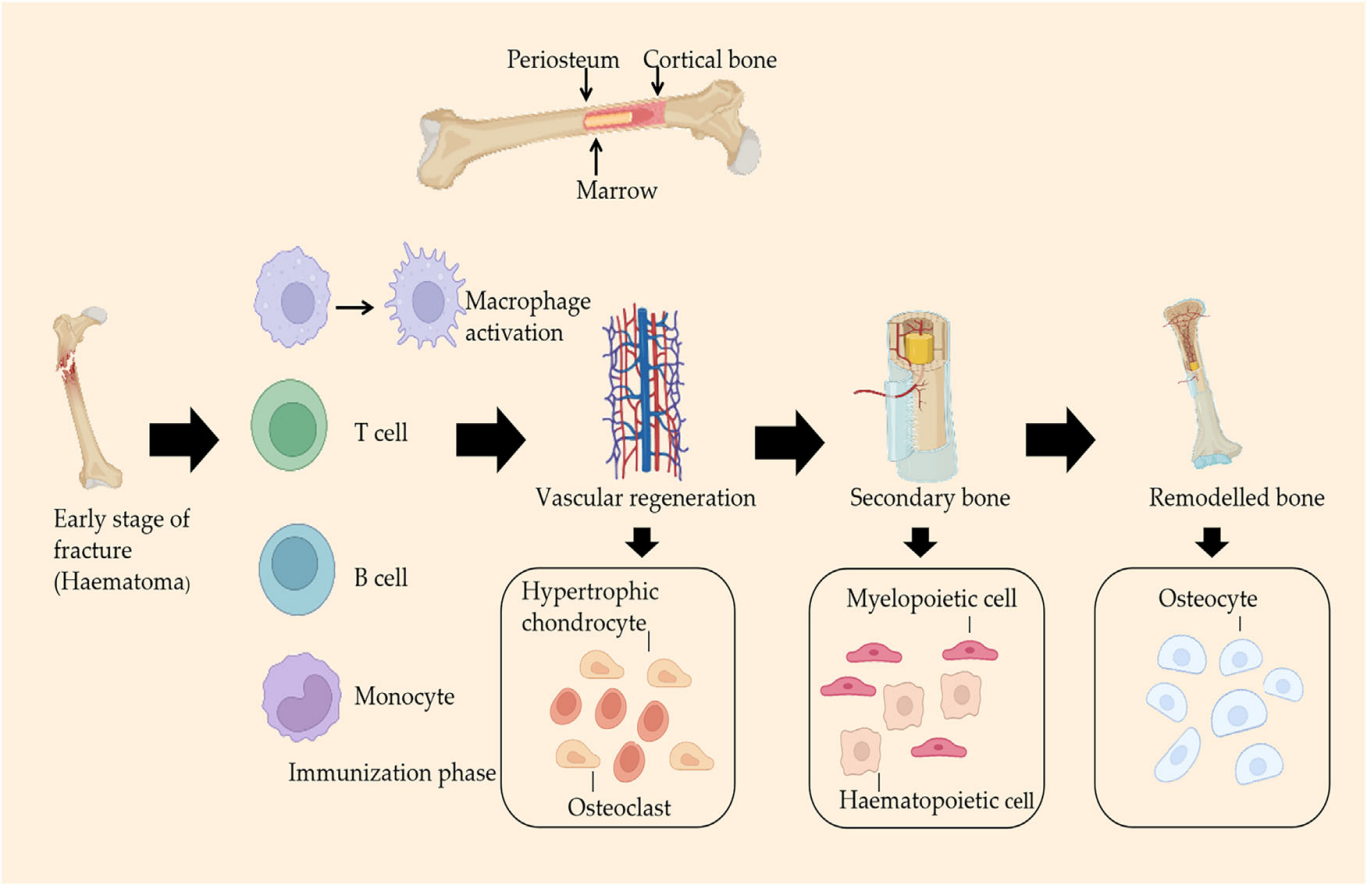
Hematoma tissue is produced at the beginning of the fracture in schematic diagram of bone tissue regeneration, followed by an immune response. This process involves macrophages, neutrophils, T cells, and B cells, forming new blood vessels and bone. Finally, healing tissue is formed.

In summary, stem cell exosomes are involved in the whole process of skin tissue regeneration and effectively regulate the inflammatory response. They cause proliferative differentiation of endothelial fibroblasts, which results in neoangiogenesis and the inhibition of scarring (Figures 1 and 2).

### 3.2 Stem cell exosomes promote bone regeneration

The regeneration of bone defects caused by trauma, infection, tumors, or inherent genetic diseases is a global clinical challenge. More than 10 million bone graft procedures are performed each year, and this number is growing at a rate of 10% annually.^189^ Bone has a certain ability to regenerate; however, once the critical size is exceeded, spontaneous regeneration of bone is limited. Therefore, a substance is needed to promote bone regeneration. Bone repair and regeneration are complex processes, including tissue regeneration, angiogenesis, and immune regulation, which not only involve BMSCs, osteoblasts, osteoclasts, bone precursor cells, and other bone‐related cells but also immune cells, vascular endothelial cells, and other system cells also play important roles.^190^ Exosomes are tiny vesicles secreted by living cells^191^, ^192^ that carry relevant proteins, lipids, nucleic acids, and other substances derived from late endonucleosomal multivesicular bodies, which play an important role in intercellular information transfer.^193^, ^194^, ^195^ Exosomes are products of the paracrine mechanism of stem cells, are more rapidly utilized than stem cells themselves, and have a wide range of clinical applications.^196^, ^197^, ^198^ Regev‐Rudzki N et al.^194^ examined the therapeutic effects of MSC‐Exos during fracture healing in a CD9−/− mouse model and found that MSC‐Exos were effective in promoting fracture healing. Zhang et al.^199^ reported results of implanting human embryonic‐derived exosomes in a rat model of cartilage defect and found that exosome treatment resulted in the complete recovery of cartilage and subchondral bone by the 12th week. These two studies demonstrated the efficacy of stem cell exosomes in bone regeneration laying a foundation for subsequent studies, provided a guiding role, and showed potential of the stem cell exosomes for clinical application for bone regeneration.^200^ Thus, stem cell‐derived exosomes have become a current research hotspot for tissue injury repair.

In the early stages of bone injury, the acute response to trauma produces a hematoma, preventing further serious trauma such as fractures.^201^ An immune response ensues with the secretion of IL‐1, IL‐6, and TNF‐α by neutrophils and macrophages during the first 24 h after the fracture, helping to initiate the repair process through cell recruitment.^202^ An important immunomodulatory property of stem cell exosomes is the promotion of M2‐type macrophage polarization and enhanced expression of anti‐inflammatory cytokines, thereby reducing the local inflammatory response. This immune response is critical for the overall repair process. Lo Sicco et al.^203^ reported that adipose stem cells can secrete exosomes with anti‐inflammatory effects when cocultured with macrophages and are effectively internalized, promoting macrophage proliferation and enhancing their polarization from M1 to M2 type in a short period of time. They also showed that MSC‐Exos can effectively reduce the local inflammatory response in mice with skeletal muscle injury in in vivo experiments.^203^ This study confirmed the ability of MSC‐Exos to regulate macrophage polarization, which may have potential applications in skeletal muscle tissue repair and regeneration. In an immunocompetent rat model, human embryonic MSC‐Exos promoted chondrocyte proliferation and matrix synthesis, with more M2‐type macrophage infiltration than M1‐type macrophages at cartilage defects, along with the reduced expression of inflammatory cytokines IL‐1β and TNF‐α, thereby promoting cartilage repair.^204^ Zhang et al.^205^ examined the role of MSC‐Exos in the regulation of the inflammatory response, nociceptive behavior, condylar cartilage and subchondral bone healing in an immunocompetent rat model of temporomandibular joint osteoarthritis. The observed exosome‐mediated repair of osteoarthritic temporomandibular joint osteoarthritis was characterized by the early inhibition of pain and inflammation‐reducing degeneration, followed by sustained proliferation.^205^ The results showed progressive improvement in matrix expression and subchondral bone structure in rats, leading to overall joint recovery and regeneration. Therefore, stem cell exosomes can promote tissue regeneration by modulating immune function, providing a basis for cell‐free therapy. However, the mechanism of action of stem cell exosomes still requires thorough investigation before clinical trials can be conducted. Stem cell exosomes produce growth factors such as TGFβ, BMPs, IGF‐1, PDGF, FGF2, and VEGF and chemokines such as monocyte chemotactic protein‐1 and monocyte inflammatory protein‐1a by paracrine action, all of which contribute to the stimulation of neoangiogenesis at the fracture site. Takeuchi et al.^206^ implanted human bone marrow‐derived MSC‐Exos in a rat cranial defect model. MSC‐Exos enhanced cell migration and the expression of osteogenic and angiogenic genes in MSCs, compared with that in other groups by the fourth week after treatment. Histologically, the MSC‐Exos group showed significant aggregation of osteoblast‐like cells and vascular endothelial cells.^206^ This study illustrated that MSC‐Exos promote angiogenesis and bone regeneration at an early stage. Considering the tissue regeneration of transplanted cells and their secretome it was concluded that exosomes might play an important role, especially in angiogenesis. Another study showed that transplantation of UMSC‐Exos significantly enhanced angiogenesis and bone healing in a rat model of a femoral fracture.^207^ Implanted UMSC‐Exos may be a key clinical strategy for accelerating fracture healing by promoting angiogenesis. Another study determined that huc‐mscs‐sev can promote osteogenesis and angiogenesis by delivering miR‐23a‐3p to activate the PTEN/AKT signaling pathway for vascularized bone regeneration.^208^ This study described the key issues associated with bone regeneration and reconstruction. In mouse and rat models, hard healing tissue remodeling begins 3−4 weeks after fracture and continues for months to years.^209^ As a cell‐free agent, stem cell exosomes avoid both the immune rejection of stem cell therapy and ethical controversy and reflect a better prospect for application in tissue regeneration. As nanoparticles (NPs), exosomes can cross various barriers (e.g., blood‐cerebrospinal fluid barrier, capillaries, etc.) and can be directly taken up by and act on target cells with higher action efficiency. Exosome therapy can effectively avoid safety issues associated with the direct application of stem cell therapy and is easy to prepare and transport. However, the mode, dose, and long‐term safety of its application in humans have yet to be evaluated. As the mechanisms of action of stem cell exosomes are studied further, their effects and mechanisms on tissue regeneration will become clearer.

## 4 NOVEL NANOFORMULATIONS LOADED WITH STEM CELLS AND EXOSOMES

With the continuous advancement in nanotechnology, combining stem cells and exosomes with nanotechnology makes treatment more precise and effective. Nanotechnology can allow stem cells to be targeted to specific locations and can also help stem cells in applying their therapeutic potential to treat, heal, and repair damaged tissues in an effective and safe manner.^210^, ^211^ Nanotechnology can solve the abnormal proliferation and differentiation of stem cells during the treatment process,^212^ improves the possibility of reducing the limitations of stem cells in the treatment of cell regeneration in injured or degenerated organs, enhances tissue repair, and assists in cellular reconstruction. Tissue engineering combined with nanotechnology can also support the growth of stem cells.^213^ To exploit the potential of stem cells for biotherapeutic applications, nanotechnology offers the ability to control the size of molecules and processes that control the fate of stem cells.^214^ Many studies on exosomes have used vesicle size and biomarkers to isolate and identify exosomes, but fail to recognize exosomes properly, or make full use of them to reach the designated site.^215^ Once released, exosomes are involved in various physiological activities and some of the effective substances are lost in this process. The clinical application of exosomes as drug carriers still faces some challenges, such as the effective introduction of exosomes to the target site.^216^ Exosomal drug delivery strategies can be divided into two categories: endogenous loading and exogenous loading, which refers to the cargo molecule being loaded in the cell that produces the exosome and the extraction and purification of exosomes before completing the loading process, respectively. Common methods of exogenous loading include incubation, electroporation, ultrasound, and repeated freeze–thaw cycles. Limiting the possibility of exosomes binding to other components allows them to successfully reach the specified location. It is necessary to achieve a combination of nanoagents and exosomes for effective clinical application.^217^ The combination of nanoagents and exosomes can solve the problems related to the exosomes delivery and their transmission to the specified location. The traditional cell culture method is susceptible to external environmental influences; the cultures require extremely specialized conditions and are unstable, leading to the wastage of resources. When exosomes were discovered in the 1980s, they were called the “trash” of cellular metabolism. Combining exosomes with nanoformulations allows resources to be repurposed, which is also an excellent material for drug delivery and tissue regeneration.^218^ Using nanotechnology to adsorb or encapsulate stem cells and exosomes in nanomaterials can improve drug stability and targeting. Specific types of nanoformulations include polymeric nanoformulations, liposomes and nanoliposomes, polymeric micelles, microspheres, and hydrogels.^219^ Several nanoagents that are commonly used for tissue regeneration are listed in Table 5.

**TABLE 5:** Nanoformulations commonly used in tissue regeneration.

Nano preparation	Material science	Structure	Slow and controlled release performance	References
Polymeric nanoformulations	Polyester Polycyanoacrylate (PBCA, PiBCA) Amphiphilic block copolymer (Mal PEG, mPEG) Dendrimer polymer(PAMAM) Chitosan	Spherical linear tubular Rod layered	The core‐sheath structured nanoparticles loaded with KET drug with free hydrogen bonding groups detached in water exhibited a better slow release effect with a release time up to 32 h.	^220^, ^221^
Liposome	Phospholipids: lecithin cephalin soybean phospholipid other synthetic phospholipids Cholesterol	Small monolayer liposomes Large monolayer liposomes Multilayer liposomes Polycystic liposomes	The results of the experiments on the slow release of liposomal pGH in depressed rats showed that GH/liposomes could exert good slow release in 3−4 d.	^222^, ^223^
Microsphere	Natural polymers: starch, albumin, gelatin, chitosan, dextran, etc. Synthetic polymers: polylactic acid (PLA), polylactide, polylactic acid‐hydroxyacetic acid (PLGA), polylactide glycolide (PLCG), polycaprolactone, polyhydroxybutyric acid, etc.	Solid microsphere Hollow microsphere Porous microsphere	Compared with PLA‐loaded microspheres, PLA/CoFE_2_O_4_‐loaded microspheres released more slowly during the test cycle, with a cumulative percentage release of 62.40% at 46 h, while PLA‐loaded microspheres already reached 74.02% at 24 h.	^224^, ^225^
Hydrogel	Natural hydrogels: Hyaluronic acid Collagen Sodium alginate Synthetic hydrogels Polyacrylamide Polyethylene glycol	Primary structure: Hydrophilic groups (carboxyl amido hydroxyl) Hydrophobic groups: alkyl Secondary structure: Mildly cross‐linked three‐dimensional network structure Three level structure: Amorphous structure	In simulated gastric juice, the release rate was about 10% within 0.5 h due to the release of surface drug, as the low dissolution rate in simulated gastric juice makes the release very slow.	^226^
Polymeric micelles	Hydrophilic section material: PEG, PEO, PVP Hydrophobic section materials: polypropylene, polystyrene, polyamino acids, polylactic acid, spermine, or short‐chain phospholipids	Block polymer micelles Grafted polymer micelles Polyelectrolyte micelles Noncovalent bond micelles	Targeted polymer micelles loaded with celestin showed slow release performance, with approximately 14.87% of the cumulative release of celestin released within 1.5 h and approximately 70% of XT released and equilibrated at 20 h.	^227^

### 4.1 Polymeric nanoformulations

Polymeric NPs are efficient drug delivery carriers that can be divided into synthetic and natural polymers and have many outstanding pharmacokinetic properties. The polymeric nanoformulations bind to the stem cells and help the drug reach the designated site and exert its therapeutic effect. Researchers have identified NPs as potential candidates to regulate NSC differentiation, which currently allows stem cells to differentiate into various cell types.^228^ Autologous adipose‐derived stem cells (ADSCs) can be safe and effective for the treatment of chronic myocardial ischemia and acute myocardial infarction, but neither ADSCs nor simvastatin‐coupled nanoformulations alone can achieve the desired therapeutic effect.^229^ After systemic administration of both, a small number of porous polylactide‐co‐glycolide (PGLA)‐NP‐loaded ADSCs gradually release statin for tissue regeneration,^230^ significantly enhancing the therapeutic effect. To overcome the resistance to chemotherapy drugs, researchers have designed a NP containing RGD^231^, ^232^ and increased the efficacy of adriamycin against cancer stem cells (CSCs). NPs can enhance the transplantation potential of human hematopoietic stem cells,^233^ suggesting that NPs have the potential to overcome the current limitations of HSC gene editing. Chitosan medium could be used to mimic the microenvironment of CSCs, increase the expression of stem cell characterization‐related genes^220^ and markers of CSCs to block differentiation and reduce the number of CSCs.

The combination of polymeric magnetic nanoformulations (MNPs) and exosomes provides new avenues for exosome‐based nanotherapies. Local release of exosomes captured under acidic pH conditions by accumulation of nanoformulations as well as cleavage of hydrazine bonds in the presence of a local magnetic field.^234^ By encapsulating adriamycin (DOX) into isolated exosomes, MNPs are encapsulated to allow the accumulation of exosomes at the site of the lesion and subsequently the targeted release of exosomes.^235^ In combination, the drugs can kill cancer cells. Although MNPs can deliver anti‐HIV drugs through an in vitro blood–brain barrier model, high doses of MNPs may damage cells. Correspondingly, exosomal EVs (xev) can cross the blood–brain barrier but lack long‐range site specificity. When MNPs and xevs are combined as nanocarriers,^236^ they can target delivery of anti‐HIV fusion agents effectively across the blood–brain barrier. Both MNPs and exosomes from stem cells have been shown to be effective in wound healing. The preparation of exosomes using BMSCs stimulated by MNPs in conjunction with static magnetic fields^237^ can further enhance wound repair.

### 4.2 Liposomes and nanoliposomes

Liposomes act as nanocarriers for targeted drug delivery for both hydrophilic and lipophilic drugs and improve drug solubility and stability.^222^, ^223^ Preparation of aspirin‐containing liposomes by a thin film dispersion method and integration into DOPA3d printed PCL scaffold^238^ enhances PCL stents. It has been verified that CD146 + liposome magnetic beads could successfully separate CD146 and ADSCs,^239^ which can promote the repair of articular cartilage defects. If the liposome is injected directly into the bone cavity, TGF is released over several weeks. TGF‐loaded liposome‐coupled scaffolds exhibit enhanced release and localization. The subcutaneous implantation of hydroxyphosphate composite scaffolds coupled with bisphosphate‐encapsulated liposomes in rats exhibited a strong binding effect, which increased drug retention.^240^ Long‐circulating polyethylene glycolyzed poly (tritiated ester) liposomes induce CSCs apoptosis in mice and are effective therapeutic agents for further inhibition of CSCs growth.^241^ Cross‐linked multilayer liposomes composed of adriamycin (Dox) and salicin (Sal) interact with breast cancer tumor cells as well as CSCs and effectively inhibit the cancer cells.^242^ Biocompatible nanoprobes consisting of liposome‐indocyanine green hybrid vesicles for safe labeling of hMSCs can track cells after drug administration^243^ until the drug is fully effective.

The binding of liposomes to exosomes can improve their targeting and increase their retention time, apart from the other remarkable features. A hybrid therapeutic nanovesicle in the conjugate of exosomes, drug‐loaded thermosensitive liposomes, and hGLV loaded with photothermal agents effectively targeted homologous tumors in mice resulting in excellent photothermal treatment and complete tumor elimination.^244^ The efficiency of exosome encapsulation of large nucleic acids was largely enhanced by mixing the exosomes with liposomes. Subsequently, the synthesized liposome exosome mixture efficiently encapsulated large plasmids, including CRISPR‐Cas9 expression vectors.^245^ The instability in the transport of exosomes to tumor cells can be resolved by fusion delivery of exosomes and liposomes,^246^ which are effectively absorbed by tumor cells and then can be concentrated near the tumor to cause apoptosis.

### 4.3 Microspheres

Microspheres are the only effective means of introducing biological materials into real‐time differentiated ESC culture. Microspheres as nanoagents can track MSCs, deliver small molecules, and promote stem cells.^224^, ^225^ The encapsulation of stem cells in microlets can be used as stem cell repair units.^247^ Microspheres in stirred suspension can preserve stem cells from human ADSCs (hASCs), which bind to microspheres for a period of time after higher levels of pluripotent markers expressed by hASCs,^248^ and improve the proliferation, colony formation, network formation, multimesenchymal differentiation, and regenerative capacity of stem cells. Injection of umbilical cord‐derived MSCs microspheres with controlled particle size and cell encapsulation ability into the rat uterus^249^ indicated their nature in promoting the repair and regeneration of the endometrium. BMSCs in the presence of open porous poly (lactic‐co‐glycolic acid) microspheres can enhance cell proliferation in vitro and in vivo promoting cartilage regeneration.^250^ Sodium alginate‐based Janus microspheres for targeted delivery of MSCs to cartilage were measured and found to be less toxic to MSCs^251^ with good restorative ability.

Exosomes have isolation and detection limitations during cancer diagnosis; furthermore, progress in the studies on exosome analysis is slow. Researchers have discovered a microfluidic device‐based method for exosome isolation and detection, realizing that the microsphere‐mediated dielectrophoretic separation and immunoaffinity detection^252^ improve the effectiveness of exosomes in tumor marker detection and cancer diagnosis. Exosomes have significant regenerative potential for bone tissue engineering; conversely, the therapeutic potential of exosomes is limited, and there is some attrition during delivery. The high biological activity of exosomes adsorbed onto the surface of porous PLGA injectable microspheres with bioinspired polydopamine (PDA) coating enables the delivery of exosomes to effectively induce tissue regeneration,^253^ thus facilitating the clinical translation of exosome‐based therapies. Integration of a PLGA microsphere delivery platform into interpenetrating network hydrogels facilitates sustained delivery of MSC‐Exos, thereby promoting endogenous AF repair,^254^, ^255^ illustrates the potential of exosomes for cell‐free bioactive repair.

### 4.4 Hydrogels

The connection between hydrogels and stem cells is complex due to the involvement of various factors, such as porosity, different polymer types, stiffness, compatibility, and degradation. As a carrier of stem cells, the right type of hydrogel must be selected for encapsulation in different situations for the purpose of transporting stem cells.^226^ Hydrogel materials have good biocompatibility and biodegradability and can solve the problem of the low survival rate of stem cell transplantation after spinal cord injury (SCI).^256^ Cells encapsulated in hydrogel microcapsules containing a thin oil layer ensure high cell viability before the cells reach the area of inflammation, segmental rupture of the hydrogel with reduced stiffness in the process of movement, and delivery of UCB‐MSCs to the sites with inflammation,^257^ thus releasing the active substances to eliminate the inflammation. The use of acrylic hyaluronic acid (HA) hydrogel microspheres, a biodegradable hydrogel, can deliver carriers in vivo and release cells to local targets.^258^ A study reported that to enhance the attachment ability and preserve bone marrow MSCs in hydrogels, the viability, density, and delivery of paracrine factors in hMSCs using agarose‐carbohydrate hydrogels must be optimized.^259^, ^260^

Hydrogels efficiently deliver exosomes to individual cells, maintaining the biological activity of exosomes while performing various repairs. Hydrogels can stimulate the release of MSC‐Exos and heal better than exosomes or hydrogels alone,^261^ suggesting that sustained release of exosomes and hydrogels can synergistically promote wound healing. The synthesis of exosome gels by immobilizing exosomes in peptide‐modified adhesive hydrogels improved the retention and release of exosomes in injured spinal cord tissue.^262^ Experiments have demonstrated that hydrogels significantly improve the residence time and stability of exosomes in vivo, and hydrogel‐bound exosomes have enhanced endothelial protection and proangiogenic capacity in vitro.^263^ The combination of exosomes and hydrogels promotes wound healing, and hydrogels are effective in delivering exosomes and improving exosomal capacity.^181^

### 4.5 Polymeric micelles

Polymeric micelles are self‐assembled from amphiphilic polymers in an easy‐to‐use manner to achieve optimal loading, stability, body circulation, and targeted delivery.^227^ Dexmedetomidine micelles can be internalized by osteoblast‐like cells and rBMSCs, resulting in the release of dexmedetomidine.^264^ Ibuprofen esterase‐responsive copolymer nanomicelles further enhance the adaptability of (NPPCs) in harsh environments and enhance their repair capacity.^265^, ^266^ Polymeric micelle drugs have higher cytotoxicity and inhibition of CSC colony formation than free drugs, and can inhibit tumor growth by CSCs.^267^ Polymeric micelles assayed for their effects on stem cell proliferation and differentiation^268^ showed that they promote adipogenesis, chondrogenesis, and osteogenesis of stem cells. A new micelle‐assisted method for efficiently loading anticancer drugs into exosome‐like vesicles has been discovered with improved tumor accumulation and retention.^269^ Using the tumor characteristics of MSCs, a drug transfer system based on MSCs was developed, and paclitaxel (PTX)‐encapsulated HA poly (d, l‐lactate‐co‐glycolide) polymer gel (PTX/HA‐PLGA) micelles were used for treatment.^270^ The results showed that the overexpression of CD44 on the surface of MSCs and tumor cells not only increased the micellar load of PTX/HA‐PLGA in MSCs but also promoted drug delivery between MSCs and adjacent cells.

**FIGURE 3:**
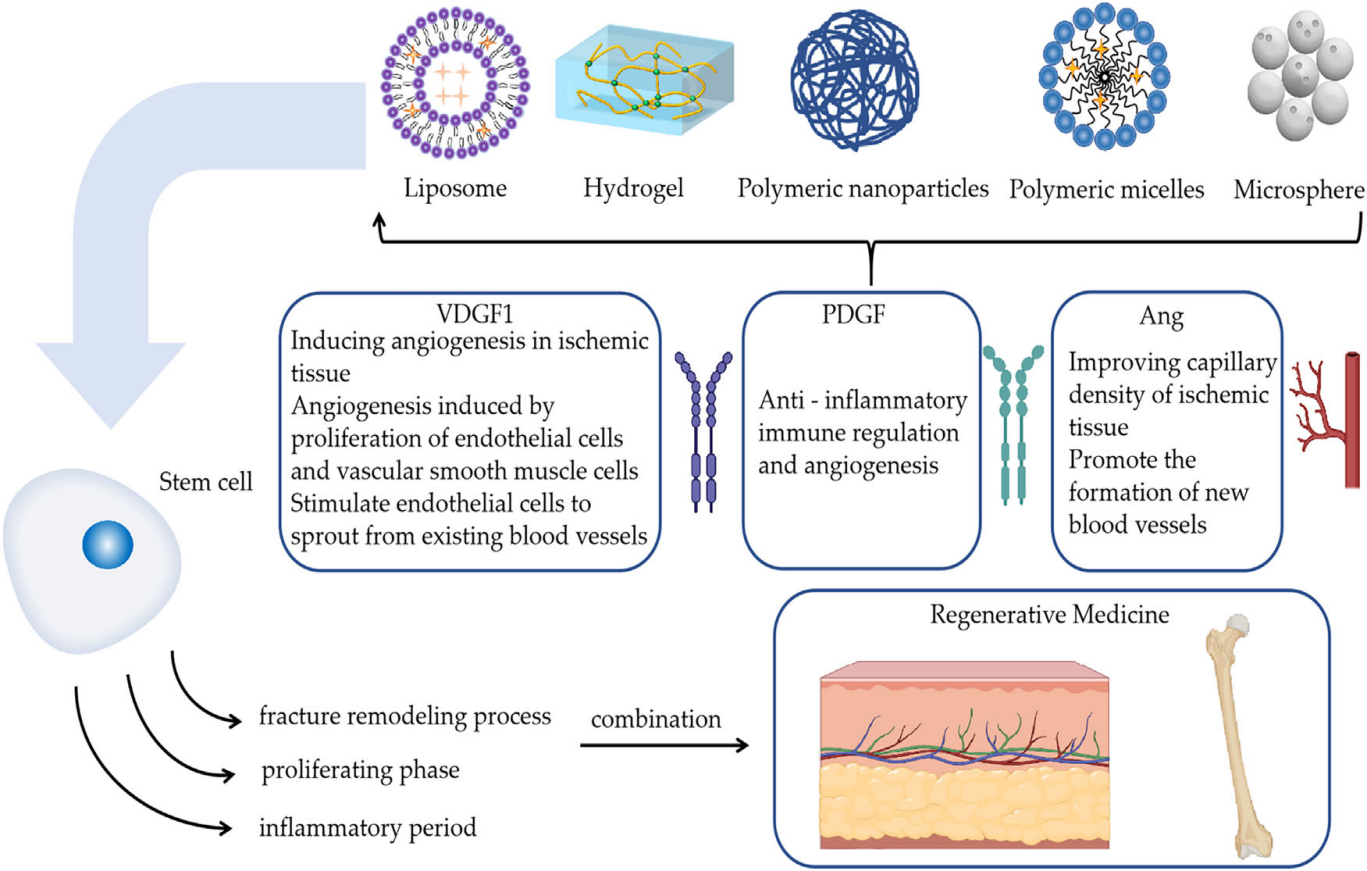
Nanopreparations of stem cells and their cytokines in regenerative medicine.

In summary, both stem cells and exosomes have some defects, such as the transport and release of a single substance. Combinations with nanoformulations can compensate for the shortcomings in this area. Nanopreparations have different effects in different fields; for example, polymeric NPs enhance the stability of drugs, especially protein‐based drugs, and have better slow and controlled release properties compared with liposomes. The interactions between nanoformulations, stem cells, and exosomes allow for more efficient transport and delivery of stem cells and facilitates exosome accumulation near a specified location, better utilization of stem cells and exosomes for tissue repair and other capabilities (Figure 3).

## 5 OTHER SYSTEMS LOADED WITH STEM CELLS AND EXOSOMES

In addition to nanotechnology, scaffolds, microneedles, and injectable hydrogel scaffolds are important for the delivery of stem cells and their exosomes. The primary aim of the stent is to provide primitive support, ensuring adhesion, migration, proliferation, differentiation, and long‐term survival of stem cells. Microneedling (MN) is a new type of biomaterial loaded with stem cells, allowing them to enter into damaged tissues with greater precision and effectiveness. The new hydrogel can effectively deliver stem cells and enhance their viability, enabling better attachment of stem cells to the scaffold.

### 5.1 Scaffolds

Due to the hostile nature of the pathological microenvironment, specific conditions must be maintained to improve their function. During the transplantation process, the scaffold acts as a bridge, a guide for axonal growth, and a carrier for the transfer of stem cells, thus changing the microenvironment. Currently, neural and MSCs have been transplanted into biomaterial scaffolds, and experiments are underway to regenerate the spinal cord.^271^, ^272^ Natural and synthetic biomaterials provide a controlled microenvironment for cells, thereby accelerating their growth and differentiation, and enhancing their viability in the human body. Biological scaffold materials are primarily used to repair damaged or missing tissues and to reconstruct their functions. Structural reconstitution and good clinical outcomes have been reported using biological scaffolds by the mechanism of release or production of impact factors, incorporation of endogenous stem cells into the scaffold, and modulation of innate immune responses.^273^ The most common method of culturing stem cells is the two‐dimensional plastic technique. This medium does not exhibit a good stem cell niche in humans; it is a very fine microenvironment that includes supporting stromal cells, the ECM, and growth factors. Therefore, researchers and clinicians are seeking the best stem cell formulation that can be used for research and clinical applications using three‐dimensional technology. Three‐dimensional culture technology can better simulate cell–cell and cell–matrix interactions.^274^ In addition to cells, different matrices and scaffolds can also be used to support complex tissues. The availability of modern technology can facilitate the expansion of cell and scaffold technology, thus providing a powerful tool for regenerative medicine.^27^In trauma or spinal fusion, scaffolding is the main bone repair method, combined with active molecules and stem cells. Cell‐based bone repair techniques have been shown to be superior to conventional techniques. A previous study showed higher osteogenic efficiency of stem cells grown in a suitable three‐dimensional scaffold support.^4^ Research has shown that three‐dimensional biological scaffolds can support and mimic the in vivo environment and help stem cells differentiate into skeletal cells. Stem cells have a strong repair capacity during burn wound repair. Application of different types of stem cells can effectively improve wound healing. In human experiments, homing of stem cells to traumatized surfaces cause re‐epithelialization, angiogenesis, granulation, inhibition of apoptosis, and regeneration of skin attachments, which reduces infection rates. Animal studies have shown that stem cells are effective in accelerating wound healing.^276^ The application of stem cells to burn wounds can significantly promote wound healing via scaffolding. The direct implantation of three‐dimensional bioprinted stem cells into blood vessels has great potential, especially the production of organ and tissue substitutes.^277^, ^278^ Promoting the differentiation of stem cells using bioprinting technology allows scaffolds to be reshaped over a period of time.^279^, ^280^ The ability to tune bioprinting properties, which can be used to create stem cell carriers and take full advantage of cell pluripotency, is of great importance in biomaterials and bioengineering. Optimal transplantation or implantation can be achieved by combining stem cells with an artificial scaffold. The interaction between a stem cell and its scaffold has important implications for differentiation and health.^281^ Stem cells from the cystic part of rat hair were used for scanning electron microscopy, using the method of extracting collagen preparation and scaffolding. The results indicated that the growth and changes in stem cells in both scaffolded and nonscaffolded states showed clear signs of cell differentiation, displaying larger cuboidal bodies with star‐shaped nuclei. Scanning electron microscopy revealed large porosity, which facilitates cell attachment and growth.^282^ Stem cells have a significant differentiation role in osteogenesis, whereas collagen scaffolds are a suitable matrix for cell growth and differentiation. There is potential for application of injectable Matrigel as a scaffold for BMP‐transduced rat dental capsule stem cells/precursor cells (rDFSCs) to promote in vitro osteogenesis and in vivo ectopic bone formation.^283^ BMP9 significantly promoted osteogenic differentiation of rDFSCs, whereas Matrigel significantly promoted osteogenesis of rDFSCs induced by BMP. Biomaterial scaffolds combined with stem cell transplantation improve cell survival and differentiation efficiency.^284^ The use of natural and synthetic biomaterials can mimic the ECM, which promotes the differentiation of MDSCs and thus re‐establishes the correct ecological niche of muscle stem cells, positively influencing muscle repair.^285^ The binding of scaffolds with cell growth factors and ECM molecules can promote cell adhesion, proliferation, and induction of osteogenesis, thus providing signals for cell transplantation and regeneration,^286^ offering tremendous opportunities for treating a wide range of diseases.

### 5.2 Microneedling

MN is a promising new technology that can be used to provide multiple drugs for the treatment of ischemic cardiomyopathy. By encapsulating cardiac stromal cells in fibrin gels and inoculating them on the MN matrix, polymeric MNs create “channels” between MNs and the host myocardium, allowing them to deliver regenerative factors to the damaged myocardium thereby accelerating cardiac repair.^287^, ^288^ The activation of hair follicle stem cells (HFSCs) can accelerate the regeneration of hair follicles, but difficulties remain in improving and managing them effectively. A keratin microneedle can be separated from the hair for the continuous delivery of HFSC‐activated substances through the microneedle tip.^289^, ^290^ Studies have shown that microneedle devices, in combination with MSC‐derived exosomes and small‐molecule drugs, are effective in reducing doses and improving treatment efficiency. Exosomes derived from MSCs have shown promising results for the treatment of SCI. However, conventional two‐dimensional culture methods result in stem cell loss from MSCs, which greatly limits the potential efficacy of in vitro secretions from MSCs. The three‐dimensional exosome in vitro culture method is effective. Conventional exogenous drug therapy relies on repeated local injections, which cause secondary damage and are ineffective. Researchers have developed a three‐dimensional exohydrogel hybrid microneedle array patch for the in situ repair of SCI.^291^ It was shown that three‐dimensional cultured bone marrow MSCs can maintain their stem cell properties, thus three‐dimensional exosomes can effectively reduce SCI‐induced inflammation and glial scarring. A novel core–shell HA MN patch with iron‐MSC‐derived artificial nanovesicles (Fe‐MSC‐NVs) and polydopamine NPs (PDA NPs) was encapsulated in a needle tip for wound healing. Fe‐MSC‐NVs contain multiple therapeutic cytokines encapsulated in the HA nucleus at the end of the MN to promote angiogenesis.^292^ In vivo experiments have shown that Fe‐MSC‐NVs/PDA MN patches are effective in wound repair in patients with diabetes. Skin MN accelerates the process of skin regeneration by creating a large amount of microdamage, which occurs when the skin is punctured by a very fine needle. To accelerate postoperative regeneration or to improve efficacy, MN is combined with drugs, vitamins, or stem cells.^293^ Owing to its high efficacy, few side effects, and short recovery period, MN has been widely used in clinical practice. MSCs are injected by encapsulating them in biomaterials, thus enhancing their stability. However, because of its poor efficacy, numerous cells are required for treatment. In addition, injection to the target site achieved using conventional syringes by local injection can cause significant damage. Researchers have developed a separable hybrid microneedle library (d‐HMND) cell delivery system^294^ that can effectively inject MSCs locally. Researchers have invented a detachable microneedle patch, whose main component is chitosan lactate (CL) with adipose stem cell exosomes (EXO). CL and EXO synergistically promote hair regrowth and regulate the circulation of hair follicles.^295^ Animal studies have shown that drug‐free microneedle patches significantly promote hair regrowth within 7 days, compared with the typically administered minoxidil at lower doses.^296^, ^297^ Microneedles deliver stem cell secretomes directly to the site of injury, demonstrating the relative therapeutic benefits of live cells while avoiding potential limitations.^298^ To improve the efficacy, a variety of biomaterials have been developed for the continuous and controlled delivery of stem cell secretomes. Direct delivery of stem cells and therapeutic macromolecules to the cardiac tissue and vascular smooth muscle cells^299^ breaks through the nonpermeable barrier and allows the drug to go directly to the target area for maximum effect, thereby reducing the required dose. In recent years, MSCs and MN techniques have been widely used to delay aging. To investigate the effect of human umbilical cord tissue matrix (hUC‐MSC‐CM) on skin brightness and rejuvenation, researchers conducted a clinical trial in which HUC‐MSC‐CM in combination with MN was found to delay aging and could be applied to facial rejuvenation by evaluating the volunteers’ skin radiance and texture as well as their self‐satisfaction.^300^

### 5.3 Hydrogel scaffolds

Recently, applied ADSCs have been investigated, and biologically active injectable hydrogels have been developed as cell‐transfer carriers. Hydrogel scaffolds are promising regenerative materials that can mimic the ECM and deliver signaling molecules.^301^, ^302^ The bionic injectable hydrogel mimics the natural microenvironment and provides the correct biochemical information for tissue regeneration.^303^ Researchers have developed amniotic tissue hydrogels as a delivery system for adipose MSCs and retained the stem cells at the target site, which could serve as a potential stem cell carrier that could be used and developed as a potential therapeutic agent for osteoarthritis.^304^, ^305^, ^306^ ADSCs have better anti‐inflammatory, chondroprotective, and paracrine functions that can be enhanced by genetic alterations. Direct cell transport without matrix support usually results in poor survival of the treated cells. Researchers have used bone grafting via genetically engineered ADSCs.^307^ An injectable ECM‐mimetic hydrogel was developed as a cell‐transfer vehicle to create a favorable microenvironment for ADSC spreading and proliferation.^308^ Hydrogels that mimic the ECM can reduce cell death during and after injection. Currently, self‐healing natural hydrogels suffer from poor stiffness, low healing efficiency, poor biocompatibility, and poor hydrolytic stability in the treatment of SCIs. Researchers have developed an injectable self‐healing HA gel based on multiple dynamic covalent bonds.^309^ This injectable, self‐healing HA hydrogel is a promising material for nerve repair. Targeted transport of stem cells using scaffolds, microneedles, and injectable hydrogels can effectively repair lesion sites and maintain stem cells.

## 6 COMBINED APPLICATION OF GENETIC ENGINEERING TECHNOLOGY AND STEM CELL EXOSOMES

Stem cells have multiple functions, including migration, differentiation, and secretion of multiple therapeutic molecules such as immunomodulatory factors.^142^ Therefore, stem cell therapy has been used in preclinical and clinical trials and has been shown to have great potential for application in the treatment of various diseases. Recently, stem cells have been modified to enhance their innate abilities and provide new functions that allow them to be used in different biomedical fields. There are many different types of modified stem cells; for example, engineered stem cells include genetically modified stem cells for gene transfer, NP loading and delivery, and small‐molecule drug delivery.^310^ Stem cell therapy has become an effective treatment option. For example stem cell therapy has be‐come an effective treatment option for tissue regeneration. . To maximize the potential of stem cells, it is necessary to modify their properties. Genetic engineering is particularly notable in this area because of the vast array of tools available to induce gene expression in a precise and controlled manner.^311^ Genetic engineering, also known as gene splicing technology and DNA recombination technology, uses the principles of molecular genetics and modern techniques from molecular biology and microbiology to construct hybrid DNA molecules in vitro from genes of different origins according to a specific structure and then transplants them into living cells to achieve the genetic characteristics of organisms, obtain new varieties, and produce new products.^312^, ^313^ Cell‐based bone regeneration therapy has been shown to be a better therapeutic choice because it reduces immune risk and bodily pain. Clinical studies have shown that different types of stem cells, especially MSCs, can effectively treat a variety of bone‐related diseases.^314^ The clinical challenges in bone regenerative medicine have led to the development of cell and tissue engineering.^315^ In particular, the development of molecular biology has laid the foundation for designing genetic strategies for bone tissue repair. Therefore, genetic engineering is an effective way to achieve continuous cell differentiation and ECM production. Gene therapy is a traditional method for improving the regenerative capacity of bone tissue. Recent studies have shown that genetic engineering techniques have become important tools for establishing effective bone, cartilage, and connective tissue.^316^

Natural stem cells isolated from humans have been used for therapeutic treatments for decades. This includes the transplantation of primary cells such as hematopoietic stem cells, MSCs, and more recently, pluripotent stem cell derivatives.^317^ However, with the development of cell engineering technologies, the new generation of stem cell‐based therapies will greatly expand their application. For example, stem cells improve drug and tumor lysis virus delivery to recalcitrant tumors and convert them into angiogenic, neurotrophic, and anti‐inflammatory factors, thereby accelerating the healing of injured or damaged tissue.^318^ Advances in reprogramming technologies have led to the emergence of induced pluripotent stem cells (iPSCs). Thereafter, the progressive research has led to the development of various effective methods that enable iPSCs to differentiate into various cell types, thus providing new avenues for disease models.^319^ In fact, cells derived from iPSCs have been widely used to study the molecular and cellular pathophysiological mechanisms underlying genetic diseases. However, the greatest difficulty in current research on iPSC cell‐based disease models is to identify the role of disease‐causing genetic variants. In the last decade, researchers have greatly improved genome editing technologies, one of which is clustering rule interval short palindromic repeat related system 9 (CRISPR/Cas9). This technique efficiently modifies a specific, discontinuous protein with a small guide RNA molecule.^320^, ^321^ This technology allows for the creation of isogenically regulated or mutated cell lines, thus focusing on the study of pathologies due to specific variants.^322^ Combining state‐of‐the‐art tissue engineering with CRISPR/Cas9‐mediated genome engineering allows the study of hPSCs in kidney diseases with unprecedented potential. In recent years, genetically engineered mutations using CRISPR/Cas9‐mediated mutagenesis have been shown to replicate polycystic kidney disease and glomerular disease phenotypes.^323^ Genetic or molecular engineering of exosomes enhances their target specificity and anticancer activity, with low toxicity.^324^ The modification of cultured cells using genetic engineering techniques in vitro can play an important role in the treatment of diseases. After the isolation and purification of exosomes, ncRNAs are loaded with mimics or inhibitors to modify the exosome surface and enhance targeting ability.^325^ Exosomes from stem cells have been widely used as “cell‐free” therapies to promote the regeneration and reconstruction of various tissues. However, resource constraints and a lack of effective therapeutic outcomes have limited the use of exosomes. Stem cell‐mediated gene therapy could create exosomes that promote bone regeneration. MSCs use protein‐2 gene modification to alter exosome content, promote bone repair, and make them more biocompatible and potentially clinically useful.^326^ A hybrid exosome‐based nanoscale delivery vehicle consisting of engineered exosomes and liposomes fused together can enable the selective encapsulation and delivery of CRISPR/Cas9 plasmids to chondrocytes embedded in articular cartilage, thereby reducing cartilage damage.^327^

## 7 CONCLUSIONS AND OUTLOOK

One of the main goals of tissue engineering and regenerative medicine is to restore damaged skin and bones. The issues of skin tissue and bone healing regeneration are gradually gaining attention and have become research hotspots. Recent studies have demonstrated the significant potential of stem cells in the fields of skin regeneration and bone healing. Wound healing is complex, and stem cells can simultaneously inhibit and increase inflammatory and anti‐inflammatory factors during wound repair, respectively.^302^

Stem cells and their exosomes play different roles at each stage of skin and bone tissue healing to promote tissue repair. Despite the advantages of stem cell therapy, there are limitations to its use as a single substance for therapeutic purposes. Stem cells are classified as ESCs or adult stem cells (AS) according to their developmental stage. Currently, the directed differentiation of human ESCs, immune rejection caused by clinical application, and lack of sources of human ESCs have limited the rapid and widespread use of ESCs in clinical practice.^328^ From the perspective of clinical application, AS cells are superior to ESCs, and self‐AS cell transplantation, such as bone marrow stem cells, is currently the ideal choice for clinical application.^329^ AS cells have some disadvantages, such as extremely small content, unclear specific markers, and slow division.^330^ Therefore, the isolation, purification, identification, and in vitro culture expansion of AS cells are very difficult.^331^ However, the benefits of stem cell therapy remain controversial. Therefore, a deeper understanding of endogenous repair mechanisms and their interactions with stem cells is required.^6^, ^332^ Exosomes also have problems, such as difficulty in extraction and poor retention. Therefore, we propose the formation of stem cells and stem cell exosomes into nanopreparations to provide a new way to promote skin tissue regeneration. Although this technology is relatively new, it shows great potential in stem cell research.^333^ Stem cell exosomes have anti‐inflammatory, antiaging, and wound‐healing skin tissue regeneration effects.^171^ In skin regeneration, stem cells can promote cell growth and angiogenesis and reduce inflammatory responses in areas of skin damage. It is the most important link in the process of tissue regeneration, promoting new collagen and elastic fibers, inhibiting metalloproteinase activity, and anti‐UV aging.^334^ The use of stem cells in the bone healing process greatly facilitates the speed and effectiveness of healing. The combination of nanotechnology and stem cells can maximize the therapeutic effects of stem cells. Nanoformulations can target and transport stem cells to specific sites and prolong the residence time, which not only improves the targeting of stem cell therapy but also reduces the adverse effects and can enhance the therapeutic effect and safety of stem cells to a great extent.^335^, ^336^ Despite numerous interesting studies, there is currently no uniform standard to describe the sequential manipulation of cells to obtain exosomes for clinical use. Exosomes can change the microenvironment; however, the microenvironment can also change the composition of exosomes.^337^ The advantages of exosomes in tissue repair and regenerative medicine include safety and longevity. Exosomes can stimulate the proliferation and migration of senescent human dermal fibroblasts and their therapeutic potential should be explored.^338^ The combination of nanoagents and exosomes can treat tissue damage caused by inflammation and prolong residence time at specific sites.^339^ Nanoformulations also have some disadvantages. Polymeric NPs have disadvantages, such as a higher risk of particle aggregation and immunogenicity‐related toxicity. Therefore, very few polymeric NPs have been approved for clinical use. Liposomes also have some problems in application, such as low encapsulation rate, rapid release of water‐soluble drugs in the blood, and low storage stability. Drug‐carrying microspheres also have limitations, such as limited use, high price, and low drug loading. Hydrogels also have limitations; the mechanical properties of hydrogels with good lubrication are poor, and improved mechanical properties do not result in good lubrication properties. The practical applications of hydrogels in the field of articular cartilage replacement materials are limited. Polymeric micelles also have limitations such as low drug‐loading capacity, poor stability, and weak microenvironmental response. Currently, the study of stem cell exosomes is an active area, and advances in the development of technologies and related studies may provide valuable information for revealing the heterogeneity and biological functions of exosomes and advancing the development of stem cell exosomes in medical therapy and diagnosis. Future research is expected to provide new insights into the role of exosomes in physiological processes, such as tissue regeneration, organ degeneration, and aging.^340^, ^341^ The combined application of stem cells, exosomes, and nanopreparations has become a new trend in the field of skin tissue regeneration; however, many research questions remain unanswered, such as how to track the technology more accurately and consistently to explore the growth of stem cells in the body, the number of cells needed for treatment, and whether stem cells remain in the lungs after whole‐body transplantation.

Research related to the use of stem cell exosomes as drug delivery vehicles is also a hot topic, and drug delivery efficiency is another challenge.^342^ Owing to the structural diversity of exosomes, their initial structural configuration can be altered using a variety of means, such as genetic engineering, chemical procedures, physical techniques, and microfluidics, thus increasing the load of exosomes and expanding their biomedical use. In targeted therapy, exosomes have great potential to overcome the limitations of conventional NP technology.^343^ Future research will require the development of efficient techniques for loading sufficient doses of drugs into stem cell exosomes while maintaining their physical integrity and biological activity.^344^ It is believed that in the future, researchers and medical professionals will be able to overcome these challenges as their research progresses and bring safer and more efficient stem cell transplants, giving hope to patients. With the continuous development of research and regenerative medicine, stem cells as an important new star in the field of regenerative medicine will continue to contribute to the progress of medicine.

## AUTHOR CONTRIBUTION

Y. J. conceived and drafted the manuscript, drew the figures, and discussed the concepts of the manuscript. S. L. conceived and drafted the manuscript, and discussed the concepts of the manuscript. Q. Y. provided valuable discussion and funding. T. C. and D. L. discussed the concepts of the manuscript. Y. J., S. L., and Q. Y. provided valuable discussion and revised the manuscript. All authors have read and approved the final manuscript.

## CONFLICT OF INTEREST STATEMENT

The authors declare no conflict of interest.

## INFORMED CONSENT STATEMENT

Not applicable.

## ETHICS STATEMENT

Not applicable.

## Data Availability

Not applicable.
